# Non-persistence to antihypertensive drug therapy in Lithuania

**DOI:** 10.1007/s00228-022-03369-0

**Published:** 2022-08-02

**Authors:** Indre Treciokiene, Nomeda Bratcikoviene, Jolanta Gulbinovic, Bjorn Wettermark, Katja Taxis

**Affiliations:** 1 Department of PharmacoTherapy, -Epidemiology & -Economics, Faculty of Science and Engineering, University of Groningen, -Epidemiology & -Economics, Groningen, Netherlands; 2 Pharmacy Center, Institute of Biomedical Science, Faculty of Medicine, Vilnius University, Vilnius, Lithuania; 3 Department of Mathematical Statistics, Faculty of Fundamental Sciences, Vilnius Tech, Vilnius, Lithuania; 4 Department of Human and Medical Genetics, Institute of Biomedical Science, Faculty of Medicine, Vilnius University, Vilnius, Lithuania; 5 Department of Pathology, Forensic Medicine and Pharmacology, Faculty of Medicine, Vilnius University, Vilnius, Lithuania; 6 Department of Pharmacy, Faculty of Pharmacy, Uppsala University, Uppsala, Sweden

**Keywords:** Hypertension, Treatment initiation, Persistence, Real-world data

## Abstract

**Purpose:**

Poor persistence to antihypertensive therapy is an important cause of treatment failure. Investigating persistence is especially important in countries with a high cardiovascular mortality, like Lithuania. The aim of this study was to describe the antihypertensive treatment at initiation, to determine the percentage of patients not being persistent with antihypertensive treatment after 1 year and to explore factors associated with non-persistence.

**Methods:**

In this cohort study, data on dispensed prescription medicines from the Lithuanian National Health Insurance Fund (NHIF) were used. All adult patients with a diagnosis of hypertension having first antihypertensive dispensed in 2018 were included. Descriptive statistics was used to determine the number of patients started with monotherapy and combination therapy. Treatment choice by Anatomical Therapeutic Chemical (ATC) and number of active pharmaceutical ingredient (API) was described. Non-persistence was assessed using the anniversary method. Multivariate logistic regression was used to explore factors associated with non-persistence.

**Results:**

A total of 72,088 patients were included into the study, 56% started on monotherapy treatment, with 49% being dispensed an angiotensin converting enzyme inhibitor, and 44% started on combination therapy. Overall, 57% of patients were non-persistent after 1 year. Patients’ gender and prescriber qualification showed no association with non-persistence. Younger patients, patients from rural area, patients started with monotherapy, and patients with no medication change had higher odds to become non-persistent.

**Conclusions:**

The majority of patients were initiated with treatment following hypertension management guidelines, but it is of concern that over half of the patients were non-persistent to antihypertensive therapy in the first year.

## Introduction

Cardiovascular diseases (CVD) are the leading cause of death, accounting globally for 32% of all deaths in 2019 [1]. In Europe, data from 2016 shows that CVD accounts for 45% of all deaths, despite the fact that the age-standardized CVD mortality rate is decreasing in all the countries [2]. Lithuania, with 56% of deaths due to CVD, is among the European countries like Latvia, Romania, and Bulgaria with the highest mortality due to CVD [3]. Hypertension is considered the number one risk factor for mortality and morbidity due to CVD and is considered one of the most prevalent chronic diseases in Lithuania [4]. Official statistics show that around 32% of the adult population had a diagnosis of hypertension in 2020 [5]. Management of hypertension is recognized as the most effective way to prevent target organ damage and to reduce the cardiovascular mortality [6]. But a study by Lisauskiene from 2021 suggested wide under treatment in Lithuanian. This drug utilization study in cardiovascular patients observed 9% and even 21% less cardiovascular medicines use in Lithuania compared to Sweden and Norway, respectively. Furthermore, cardiovascular age-standardized death rates were twice as high in Lithuania compared to those Scandinavian countries [7].

Worldwide, guidelines recommend early diagnosis of hypertension, lifestyle modification, and initiation and continuation of pharmacological treatment to decrease mortality and morbidity due to CVD [6, 8]. The National Lithuanian High Cardiovascular Risk (LitHiR) program was launched in 2006. The program aimed to detect and manage cardiovascular risk factors including early detection of hypertension [9]. This program, promoted via mass media, enrolled men between 40 and 54 years and women between 50 and 64 years without known CVD. Results suggest an improvement in hypertension control from 20% of patients in 2009 to 25% in 2018 [10].

Hypertension management guidelines by European Society of Cardiology recommend two approaches to initiate antihypertensive treatment [6, 11]. One approach is initial monotherapy followed by the addition of a second, a third, and further antihypertensive, in case of lack of control, commonly known as step-care treatment. The other option is in particular recommended for patients with an initial blood pressure > 150 mmHg who require reduction of > 20 mmHg. For those patients, a combination of two different antihypertensive drug classes is favored as initial treatment [11]. Medicines from five groups are recommended; angiotensin-converting enzyme inhibitors (ACEi), angiotensin II receptor blockers (ARBs), diuretics, beta blockers, and calcium channel blockers. Drug utilization research from around the world has shown that in fact monotherapy was the preferred option to initiate antihypertensive treatment in some countries [12, 13]. In others, the proportions of patients with monotherapy and combination therapy as initial treatment were similar [14] or the majority of initial prescriptions were combinations [15]. Initial treatment has not been studied in Lithuania in recent years. Despite the extensive evidence of benefits of antihypertensive treatment, many patients with hypertension do not reach target blood pressures [10, 16]. There are many reasons behind poor blood pressure control. As hypertension is a chronic disease which require life-long treatment therapy, persistence is important for hypertension management [6, 11]. Persistence with antihypertensive therapy ranges from 35 to 92% in studies all over the world [17–21], but this has not been studied in Lithuania.

The aim of this study was to describe the choice of therapy for initiation of antihypertensive treatment, to determine the percentage of patients not being persistent with antihypertensive treatment after 1 year and to explore factors associated with non-persistence.

## Methods

### Study design and data source

In this cohort study, data on dispensed and reimbursed prescription medicines were obtained from the information system “Sveidra” of the Lithuanian National Health Insurance Fund (NHIF). “Sveidra” contains data on all medical services and reimbursed medicines; it covers approximately 97% of entire population in the country [22]. Although the database contains all information on both outpatient and inpatient services [23], only information from the subsystem on reimbursed medicines with some insensitive information about patient and prescriber was used in this study. The reimbursed medicine subsystem contains information on all reimbursed prescriptions and covers up to 100% of the insured population. Reimbursed medicines could be sold outside this system, e.g., foreigners and privately insured patients. Out of pocket dispense in pharmacy is possible without prescription for a limited 1-month period. Reimbursed prescriptions could be prescribed for the period from one to 6 months. The medicines bought completely out of the pocket are not included in the “Sveidra” dataset, but the medicines that are partly subsidized are included. Personal patient and prescriber information was coded by NHIF.

### Study cohort and definitions

Adult patients over 18 years, being diagnosed with hypertension and having an antihypertensive medicine dispensed for the first time in the year 2018 were included in the study. A diagnosis of hypertension was defined as one of the following ICD 10 codes: I10 primary (essential) hypertension, I11 hypertensive heart disease with heart failure (congestive), and I11.9 hypertensive heart disease without heart failure (congestive). The patient was considered as newly initiated with hypertension treatment if he or she had no antihypertensive medicines dispensed between 2017.01.01 and 2017.12.31. The first dispensing date in 2018 was considered the index day. We also determined the last dispensation within 365 days of the index date. The following antihypertensives (with Anatomic Therapeutic Chemical (ATC) codes in brackets) were included: antiadrenergic agents, centrally (C02A) and peripherally (C02C) acting, diuretics (C03), beta receptor blockers (C07), calcium channel blockers (C08), angiotensin converting enzyme inhibitors and their combinations (C09A and C09B), angiotensin receptor blockers and their combinations (C09C and C09D), and combinations of antihypertensive medicines with lipid lowering agents (C10BX). The number of adult inhabitants of Lithuania at the beginning of the year 2018 was derived from official statistic portal [24]. A total of 2,305,886 adult inhabitants lived in Lithuania at the beginning of the year 2018.

### Outcome measures and factors studied

The incidence rate of initiation of antihypertensive treatment per 1000 inhabitants per year was calculated. For patients who were initiated on antihypertensive treatment, we determined the proportion of initiation with monotherapy and combination therapy. For monotherapy, we calculated the proportion of patients initiated with each drug class. For combination therapy, we calculated the number of active pharmaceutical ingredients (API) patients were initiated with.

**Fig. 1:**
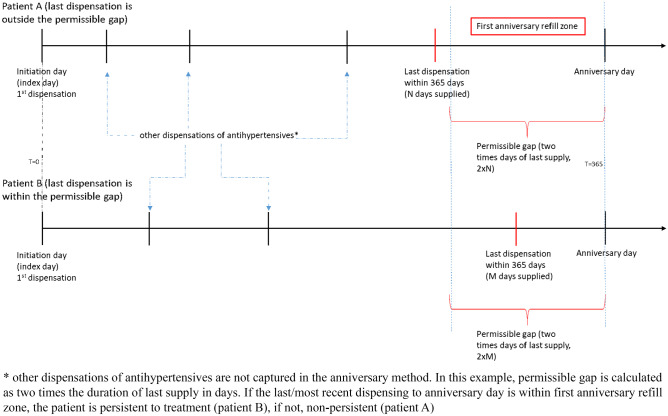
Measuring persistence with treatment anniversary method

For patients initiated on antihypertensives, we also calculated the percentage of patients who were non-persistent with antihypertensive therapy within the first year (anniversary method). Non-persistent patients were defined as patients who did not have antihypertensives dispensed at day 365 after the index date [25]. For this, a permissible gap was allowed which was defined as twice the time period of the supply days covered from the last prescription dispensed. A graphical display of the anniversary method and the permissible gap is shown in Fig. 1. Patients remaining on antihypertensive treatment, but switching between antihypertensives, were considered as persistent.

**Fig. 2:**
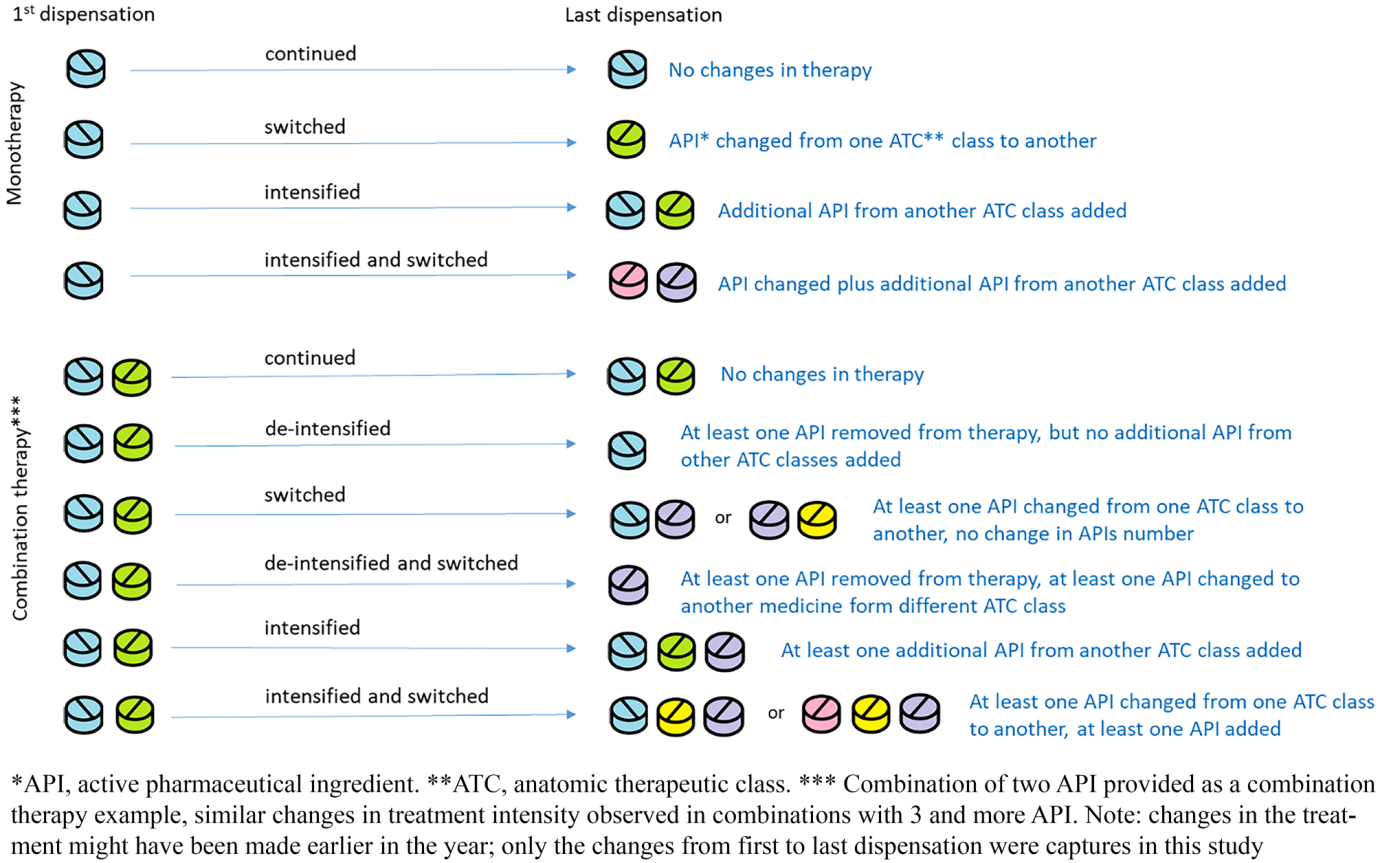
Possible differences in treatment between the 1st and the last dispensation

The following factors were tested for association with non-persistence: age in years, sex, area of primary care registration, prescriber qualification, initial treatment approach (monotherapy or combination therapy), and treatment changes after initiation. The following patient characteristics were captured at index date: sex, age in years, and patient’s primary care registration area. If the patient’s general practitioner’s location was based in a city or county center, the primary care area was considered as city. All other locations were considered as rural. Prescriber qualification was also recorded at index date and coded in 4 categories: cardiologists, general practitioner, physicians that had both qualifications of cardiologist and general practitioner, and other qualifications. We defined the following 6 categories of differences between the first dispensation and the last dispensations: continuation, intensification, de-intensification, switch, or a mix of treatment changes (Fig. 2).

### Statistical analysis

Data was processed with DBeaver SQL-based program and analyzed with IBM SPSS statistics 27. Descriptive statistics was used to describe baseline characteristics. Continuous variables were summarized using means and standard variation. Categorical variables were presented using frequencies. A stepwise multivariate logistic regression was used to explore factors associated with non-persistence. Independent continuous variables were categorized before the analyses. Multicollinearity was tested using generalized variance inflation factor (GVIF). Crude and adjusted odds ratios with 95% confidence intervals (CI) were calculated. Sensitivity analyses were performed with the permissible gap of one and a half times of prescription dispensed supply days and with no permissible gap, defined as patients who did not have a supply of medicines on the anniversary day.

Permission for biomedical research was issued by Vilnius Regional Biomedical Research Ethics Committee in 2021. Permission number 2021/2–1314˗790.

## Results

A total of 72,088 patients had a diagnosis of hypertension and received antihypertensives for the first time in 2018. This was an incidence rate of 31.3 per 1000 inhabitants. The majority of the patients with newly initiated antihypertensive treatment were over 50 years of age. Population and patients’ characteristics are provided in Table 1.

**Table 1:** Characteristics of the Lithuanian adult population and patients with hypertension that started antihypertensive treatment in 2018

		Adult population at 1st of Jan 2018 (%)	Patients initiated with antihypertensive treatment in 2018 (SD or %)	Incidence rate*
Total		2,305,886	72,088	31.3
Mean age		49.8	57.7 (SD = 14.2)	
Men		1,039,358 (45.1%)	34,257 (47.5%)	33.0
Women		1,266,528 (54.9%)	37,831 (52.5%)	29.9
Number of patients by age group	< 39	763,273 (33.1%)	6875 (9.5%)	9.0
	40–49	381,161 (16.5%)	13,078 (18.1%)	34.3
	50–59	431,278 (18.7%)	21,163 (29.4%)	49.1
	60–69	333,514 (14.5%)	16,326 (22.6%)	49.0
	70–79	238,137 (10.3%)	9279 (12.9%)	39.0
	> 80	158,523 (6.9%)	5367 (7.4%)	33.9
Number of patients per primary care registration area**	Urban	-	36,228 (50.3%)	-
	Rural	-	35,860 (49.7%)	-

^*^Incidence rate, number of new diagnoses per 1000 inhabitants

^**^Could not be derived from the official statistics for the entire population

Slightly more than half of all patients started treatment with monotherapy, the remaining on combination therapy. Most often, monotherapy was initiated with an ACE inhibitor. The majority of the medicines were prescribed by general practitioners. Treatment at initiation is provided in Table 2.

**Table 2:** Treatment at initiation

Total number of patients 72,088	Number of patients (%)			Number of patients (%)
Number of patients initiated on monotherapy*	40,591 (56.3%)	Class of medicines prescribed at initiation with monotherapy	ACE inhibitors	19,990 (49.3%)
			Beta blockers	16,084 (39.6%)
			ARB	1884 (4.6%)
			Calcium channel blockers	1559 (3.8%)
			Diuretics	747 (1.8%)
			Central acting agents	166 (0.4%)
			Peripherally acting agents	161 (0.4%)
Number of patients initiated on combination therapy**	31,497 (43.7%)	Number of active pharmaceutical ingredients included in the combination therapy	2 API	22,798 (72.4%)
			3 API	6681 (21.2%)
			4 API	1573 (5.0%)
			5 API and more	445 (1.4%)

^*^Monotherapy when medicine with one active pharmaceutical ingredient (API) is dispensed

^**^Combination therapy: when one medicine with more than one active pharmaceutical ingredients (API) or when two or more medicines dispensed; all API calculated individually; this includes fixed dose combinations with two or more API

Most of the antihypertensive treatment was initiated by general practitioner—85.4% of the initiations. Other specialists initiating treatment were cardiologist 9.9%, specialists having both qualifications of cardiologist and general practitioner 3.1%, and other qualifications specialists 1.5%. Patient received a mean supply of first dispensed medicines of 41 (SD = 25.7) days. The mean supply of the medicines which were dispensed on the date closest to the anniversary day was 64.5 (SD = 38.6) days.

### Non-persistence with antihypertensive therapy

Out of 72,088 patients who started with antihypertensive treatment, 57.4% were non-persistent within the first year after initiation. Of 40,591 patients who started monotherapy, 61.1% were non-persistent versus 52.6% of all patients who started with combination therapy. Of patients whose treatment was changed, 34.5% were non-persistent versus 67.9% of patients whose treatment was not changed after initiation. Patients whose treatment was changed and intensified adding additional active pharmaceutical ingredients (API) were most persistent to the treatment. All factors which were associated with non-persistence are provided in Table 3. Multicollinearity was rejected in logistic regression model. All explanatory variables were independent—GVIF statistics confirmed this conclusion (all GVIF were less than 1.04).

**Table 3:** Factors associated with non-persistence on antihypertensive therapy within 1 year

Characteristics		Non-persistent (%)	Crude odds ratio for non-persistence (CI)*	Adjusted odds ratio** for non-persistence (CI)*
Age, years	< 39	4567 (66.4%)	Reference	
	40–49	7713 (59.0%)	0.73 (0.68–0.77)	**0.78** (0.73–0.84)
	50–59	11,732 (55.4%)	0.63 (0.59–0.67)	**0.70** (0.66–0.74)
	60–69	8861 (54.3%)	0.60 (0.59–0.67)	**0.67** (0.63–0.71)
	70–79	5176 (57.8%)	0.64 (0.60–0.68)	**0.73** (0.68–0.78)
	> 80	3313 (61.7%)	0.82 (0.76–0.88)	**0.89** (0.82–0.96)
Gender	Female	21,601 (57.1%)	Reference	
	Male	19,761 (57.7%)	1.02 (0.99–1.06)	1.02 (0.99–1.06)
Area of primary care registration	City	20,173 (55.7%)	Reference	
	Province	21,189 (59.1%)	1.15 (1.12–1.18)	**1.16** (1.12–1.19)
Prescriber qualification	Cardiologist	4110 (57.4%)	Reference	
	GP	35,406 (57.4%)	1.01 (0.96–1.06)	0.99 (0.94–1.04)
	Both***	1282 (57.4%)	0.98 (0.89–1.08)	0.98 (0.88–1.08)
	Other	557 (57.4%)	0.76 (0.67–0.86)	0.87 (0.76–1.00)
Initiated with	Monotherapy	24,807 (61.1%)	Reference	
	Combination therapy	15,784 (51.4%)	0.71 (0.68–0.73)	**0.85** (0.83–0.88)
Difference in treatment between first and last dispensing	Continued	33,560 (67.9%)	Reference	
	Switched	2082 (39.5%)	0.31 (0.29–0.33)	**0.31** (0.29–0.33)
	Intensified	131 (32.0%)	0.22 (0.18–0.28)	**0.24** (0.20–0.30)
	De-intensified****	572 (47.6%)	0.43 (0.38–0.48)	**0.51** (0.45–0.57)
	Switched and intensified	2894 (29.2%)	0.20 (0.19–0.21)	**0.19** (0.18–0.20)
	Switched and de-intensified****	2123 (36.3%)	0.27 (0.25–0.29)	**0.32** (0.30–0.34)

Significant adjusted odds ratios in bold

^*^Calculated with 95% confidence interval

^**^Multivariate stepwise regression model including all covariates studied: age in years, sex, area of primary care registration, prescriber qualification, initial treatment approach, and further treatment strategy within 365 days

^***^Health care professional who had both cardiologist and general practitioner qualifications

^****^For initiated with combination therapy only; intensified, at least one additional API added to treatment; de-intensified, at least one API removed from treatment, no additional API from other ATC class added

The sensitivity analysis, modifying the definition of a permissible gap, showed that non-persistence was 61.8% with the permissible gap of one and a half times the period of time of the last dispensed prescription. Non-persistence was 69.1% if no permissible gap was allowed; i.e., the supply period of the last dispensed antihypertensive covered the anniversary day. The sensitivity analyses using the different permissible gaps did not change the main findings of the logistic regression (Appendix Table 4).

## Discussion

Over 3% of the entire adult population of Lithuania were initiated with antihypertensive treatment in 2018. The mean age of those patients was 57.7 years which was in the range of earlier studies, 60.3 years in 2004 and 54.5 years in 2012 in Lithuania [26], 62.0 in UK [27], 55.6 in Korea [28], and 54.5 in the USA [29].

### Treatment initiation

Slightly more than half of the patients were started on monotherapy, with the main used agents being ACE inhibitors (49%) and beta blockers (40%). An earlier study in Lithuania which covered the years 2004–2012 [26] showed that monotherapy was used for the initiation in about 75% cases in 2012, with ACE inhibitors and beta blockers as most used agents. This reduction in the percentage of patients started on monotherapy is in line with changes in the guidelines. Combination therapy is more favored in the ESC/ESH Guidelines for the management of arterial hypertension in 2018 than in 2013 [6]. Of note, we found that 166 patients were started on a central acting agent (moxonidine or rilmenidine) as monotherapy which is not recommended in any of the current hypertension treatment guidelines. Use of central acting agents is however in line with our recent study investigating drug utilization in Lithuania [30].

Over 43% of patients were started on combination treatment. Most of them were initiated with two API (72% of combination therapy patients or 32% of all patients) either in fixed or in free combinations. This is in line with the newer guidelines as highlighted above. We observed some patterns which are not in line with recommendations. There were over 2000 patients (2.8%) who were started with 4 and more antihypertensive API. This irrational approach has been observed previously in Lithuania where the number of patients who were initiated with 4 and more medicines increased from 0.5% in 2004 to nearly 2% in 2012 [26]. Eighty-five percent of the treatments were started by general practitioners indicating that hypertension treatment was started in primary care setting. Similar results were found in Denmark where general practitioners were responsible for 84% of all treatment initiation [31].

### Non-persistence

We found that 57% of patients were non-persistent to antihypertensive therapy at the end of the first treatment year. Young patients had more odds to discontinue therapy—66% of patients of 39 years and younger were non-persistent to therapy, while patients in age group of 60–69 years showed higher persistence rates—only 54% were non-persistent. Our results are at the high end of non-persistence rates reported in other studies. Around 61% discontinued initial antihypertensive treatment in Spain [32], 52% in Italy [18], 26% in Sweden [20], and 20% in Germany after 1 year [33]. A review by Cramer observed 22 studies with the non-persistence rate from 8 to 64.9%, with an average of 36.7%, after 12-month of follow-up [21]. The evidence from other countries vary and the results are difficult to compare as different inclusion criteria, different cohorts, and medication use assessment methods are used; e.g., different methods from anniversary method to refill gap method could be used to assess persistence [25].

There was no difference between women and men in the persistence to therapy at the end of first year. A systematic review and meta-analysis by Biffi et al. support our results and show no significant difference in adherence to antihypertensive treatment between men and women [34]. Patients from rural areas had a 15% higher odds on therapy non-persistence. This might show inequities in health care [35] as well as influence of education, family income, or other factors that we could not include in our study. Prescriber qualification was not associated with non-persistence. This is in contrast with a study on oral antidiabetic treatment which showed that being a physician other than a general practitioner was related to an increase of discontinuation [36].

Patients started on combination therapy were more persistent than those started on monotherapy. Confounding by indication might have influenced our results, as patients with more comorbidities or more severe disease may need a combination treatment to reach therapy targets. Those patients might be more persistent as has been found in the systematic review by Lemstra [37]. A Spanish study also found that combination therapy was linked to higher persistence rates [32]. The difference between monotherapy and combination therapy have not been extensively studied.

We found that patients who stayed on the medicines from the same initiated drug class were less persistent to therapy than patients whose initial therapy was changed. In fact, any change to treatment, either a step-care approach or change of medicines, or even de-intensification of treatment lead to a better persistence than no change. We were not able to study the frequency of visits to the doctor, so we do not know whether patients who discontinued the treatment have not returned to the doctor. It is important to note that we only included changes of the number of active pharmaceutical ingredients (API) and drug classes but not changes within the same drug class or dosage.

The main strength of this study is the large number of included patients. Our national cohort covered all patients with hypertension who claimed a first prescription of an antihypertensive medicine and thus gives a full view of worryingly high number of non-persistent patients in the first year of treatment.

The study has some limitations as well. As in all database studies using information on dispensed medicines, we do not know whether patients take the medicines as prescribed. We also could not identify patients who were prescribed antihypertensive medication, but did not have those dispensed (primary non adherence). We may have also missed antihypertensives completely paid out of pocket, but this is unlikely to be a big concern in chronic diseases like hypertension. On the other hand, the inclusion of C02A and C02C could be questioned, yet, in the cohort, each prescription had a diagnose code and those medicines were prescribed to hypertension for some patients. Our data set was restricted and no clinical information such as severity of disease and comorbidities was provided. Therefore, we could not distinguish between primary and secondary prevention as diagnoses codes could be used little differently in reimbursement data. Blood pressure, sociodemographic variables such as level of education, and income that could influence persistence [38] were not provided in the data. It might have been the case that for some patients’ discontinuation was clinically justified as the blood pressure control was reached. Patients were also not censored for being admitted to hospital or home care. Patients admitted to institutions either used their own chronic medications or could have been prescribed reimbursed medicines that have been administered for at least 1 month prior to hospitalization [39]. Some patients might have dropped out from the cohort because of death or migration, but we could not censor patients to that. This might explain the relative increase in non-persistence in the oldest age group. Finally, we followed patients for 365 days only. Studies show that non-persistent patients might return to treatment after 1 year [40] and patients with established hypertension are more persistent to therapy that newly diagnosed [41]. Further research with longer follow-up would be recommended.

Despite the methodological limitations of our study, this first investigation of non-persistence should raise concerns among clinicians and policy makers to address this clinical problem. Hypertension is highly prevalent and a life-long disease with initiation and continuation of treatment being one of the most critical interventions to decrease cardiovascular morbidity and mortality [6]. In general, most of the patients were started antihypertensive treatment following guidelines yet non-persistence with therapy could have been linked with adverse clinical outcomes and high mortality rate due to cardiovascular diseases. The Lithuanian rate is at the high end of what has been found in other studies as highlighted above. Interventions to increase therapy persistence could be done, in particular, given the high mortality rate due to cardiovascular diseases in Lithuania.

## Conclusions

The majority of patients diagnosed with hypertension were initiated with treatment following hypertension management guidelines, but it is of concern that over half of the patients were non-persistent to antihypertensive therapy in the first year. Younger patients, patients whose primary care registration was in rural area, patients started with monotherapy, and patients with no medication change had higher odds to become non-persistent.

## Data Availability

Data was obtained from the information system “Sveidra” of the Lithuanian National Health Insurance Fund (NHIF) under permission. Data are however available from the authors upon reasonable request and with permission of NHIF.

## Appendix

**Table 4:** Non-persistence and factors associated with non-persistence on antihypertensive treatment within 1 year calculated using different permissible gaps

Characteristics		Non-persistence with a permissible gap of 1,5x period of prescription dispensed supply days		Non-persistence with no permissible gap	
		Non-persistent	Adjusted Odds Ratio* for non persistence (CI)	Non-persistent	Adjusted Odds Ratio* for non-persistence
Age, years	<39	4849 (70.5%)	Reference	5236 (76.2%)	Reference
	40-49	8275 (63.3%)	**0.77** (0.73-0.83)	9183 (70.2%)	**0.79** (0.74-0.85)
	50-59	12680 (59.9%)	**0.69** (0.65-0.73)	14306 (67.6%)	**0.72** (0.68-0.77)
	60-69	9602 (58.8%)	**0.66** (0.62-0.71)	10839 (66.4%)	**0.69** (0.64-0.74)
	70-79	5589 (60.2%)	**0.72** (0.67-0.77)	6294 (67.8%)	**0.75** (0.69-0.80)
	>80	3573 (66.6%)	**0.90** (0.83-0.98)	3949 (73.6%)	0.95 (0.87-1.03) **
Gender	Female	23315 (61.6%)	Reference	26093 (69.0%)	Reference
	Male	21253 (62.0%)	1.02 (0.98-1.05)	23714 (69.2%)	1.01 (0.98-1.05)
Area of primary care registration	City	21855 (60.3%)	Reference	24542 (67.7%)	Reference
	Province	22713 (63.3%)	**1.14** (1.11-1.18)	25265 (70.5%)	**1.14** (1.10-1.18)
Prescriber qualification	Cardiologist	4408 (61.6%)	Reference	4929 (68.8%)	Reference
	GP	38166 (62.0%)	1.01 (0.95-1.06)	42637 (69.3%)	1.01 (0.95-1.06)
	Both***	1388 (61.7%)	1.00 (0.91-1.11)	1561 (69.3%)	1.02 (0.92-1.14)
	Other	606 (54.3%)	**0.85** (0.74-0.97)**	680 (60.9%)	**0.79** (0.69-0.91) **
Initiated with	Monotherapy	26513 (65.3%)	Reference	29237 (72.0%)	Reference
	Combination therapy	18055 (57.3%)	**0.86** (0.83-0.89)	20570 (65.3%)	**0.87** (0.84-0.90)
Further treatment within 365 days	Continued	35396 (71.6%)	Reference	38254 (77.4%)	Reference
	Switched	2403 (45.5%)	**0.33** (0.31-0.35)	2937 (55.7%)	**0.37** (0.35-0.39)
	Intensified	160 (39.1%)	**0.28** (0.23-0.34)	210 (51.3%)	**0.34** (0.28-0.41)
	De-intensified****	641 (53.3%)	**0.54** (0.48-0.61)	736 (61.2%)	**0.54** (0.48-0.61)
	Switched and intensified	3445 (34.8%)	**0.20** (0.19-0.21)	4523 (45.7%)	**0.24** (0.23-0.25)
	Switched and de-intensified****	2523 (43.1%)	**0.36** (0.34-0.38)	3147 (53.7%)	**0.40** (0.38-0.43)

Significant Adjusted Odds Ratios in bold

*Multivariate stepwise regression model including all covariates studied: age in years, sex, area of primary care registration, prescriber qualification, initial treatment approach and further treatment strategy within 365 days

**the Odds ratios that have changed from the main results

***Health care professional who had both cardiologist and general practitioner qualifications

****for initiated with combination therapy only; Intensified – at least one additional API added to treatment; De-intensified – at least one API removed from treatment, no additional API from other ATC class added
